# Differential associations of cooking behaviors with polycyclic aromatic hydrocarbon exposure-related platelet traits as cardiovascular risk biomarkers

**DOI:** 10.3389/fcvm.2026.1703901

**Published:** 2026-05-15

**Authors:** Quanping Yan, Yaoyao Li, Sheng Wang, Xiaohuan Yang, Ziyuan Zhang, Yinghao Yuchi, Chongjian Wang, Ge Zhao, Fuwei Xie

**Affiliations:** 1 Key Laboratory of Tobacco Chemistry, Zhengzhou Tobacco Research Institute of CNTC, Zhengzhou, China; 2 Department of Epidemiology and Biostatistics, College of Public Health, Zhengzhou University, Zhengzhou, China

**Keywords:** henan rural cohort study, platelet distribution width, platelet indicators, polycyclic aromatic hydrocarbons, quantile g-computation

## Abstract

**Introduction:**

Polycyclic aromatic hydrocarbons (PAHs) typically occur as mixtures, and their combined associations with platelet traits have been scarcely explored in rural populations. This study investigated both individual and joint associations of PAHs with platelet traits.

**Methods:**

A subpopulation (*n* = 2,124) was derived from the Henan Rural Cohort Study. Cooking and smoking behaviors were assessed by questionnaire, plasma PAH concentrations were measured using gas chromatography–mass spectrometry, and platelet traits such as mean platelet volume were obtained from routine blood tests. Individual and joint associations between plasma PAHs and platelet traits were analyzed using general linear models (GLMs) and quantile g-computation models. Furthermore, the differential associations of cooking or smoking behaviors were explored.

**Results:**

GLM showed that there were associations of certain plasma PAHs with platelet traits across participants. For instance, Dibenz(a,h)anthracene (DahA), Fluoranthene (Flu), and Pyrene (Pyr) were negatively associated with platelet count (PLT) in the total population. Positive associations of certain PAHs with platelet size indicators such as MPV and platelet distribution width (PDW) were observed. The mixture of 10 PAHs was positively associated with PDW, MPV, platelet large cell ratio, MPV/PLT ratio (MPVP), and PDW/PLT, with DahA and Benzo(a)pyrene (BaP) identified as major contributors. These associations remained among non-smoking and non-cooking men and non-smoking women who reported self-cooking.

**Discussion:**

The findings show that individual plasma PAHs and their mixture are related to increased risk of platelet dysfunction, with BaP and DahA identified as key contributors. The results indicate that the association between PAH exposure and changes in platelet indicators was prominent among women with unhealthy cooking behaviors.

## Introduction

Polycyclic aromatic hydrocarbons (PAHs) are among of the most widely distributed persistent organic pollutants (POPs) across multiple environmental media such as air, soil, and water. Due to their physicochemical properties, PAHs can accumulate far from their emission sources (1–3). The routes of human exposure to PAHs are mainly inhalation, ingestion, or skin contact (2). Accumulating evidence suggests that PAHs are associated with multisystem damage, including reproductive and cardiovascular system damage (1). For instance, associations between PAH exposure and cardiovascular disease (CVD) have been reported in different countries and populations (4–6). However, few studies have examined PAH exposure in relation to an increased risk of cardiovascular disease biomarkers, such as heart rate variability and endothelial dysfunction (7–9). Epidemiological and toxicological studies have indicated that oxidative stress and inflammation play critical roles in the development of CVDs following PAH exposure (1–3, 10). In recent years, platelets, which play a key role in the development of CVDs, have attracted increasing attention (11, 12). However, studies examining the associations between PAH exposure and platelet indices among rural populations remain scarce.

Human platelets differ considerably with regard to their size and thrombogenicity (12). Platelet size indicators, including platelet volume distribution width (PDW), mean platelet volume (MPV), platelet large cell ratio (P-LCR), and plateletcrit (PCT), reflect platelet hyperactivity and are involved in the pathophysiology of major cardiovascular events (13). Evidence suggests that platelets may serve as non-traditional cardiovascular risk factors (14). Furthermore, platelets play an important role in CVDs related to particulate matter (PM) pollution (15). Chronic exposure to low concentrations of PAHs, a classic organic component absorbed onto PM, may adversely affect platelet indicators (16–18). For instance, negative associations between PAH metabolites and PDW, MPV, P-LCR, and the MPV/platelet ratio (MPVP) have been reported (17). However, the results from one cross-sectional study showed that PAH metabolites were positively associated with PDW, MPV, and MPVP (18). The results of a longitudinal study in China suggested that chronic exposure to low concentrations of PAHs resulted in increased PDW, PLT, and MPV, with the metabolite 1-OHP showing a linear dose–response relationship with platelets (16). In addition, a study in children living in e-waste-dismantling areas reported that PAH exposure resulted in significantly higher levels of inflammatory markers, including peripheral blood neutrophil count, monocyte count, and interleukin-6, compared with levels in children from control areas (19). Similarly, Zhao found that coexposure to PAHs and phthalic acid esters was associated with elevated inflammation in children (20). However, most previous studies have used PAH metabolites in urine to assess the adverse effects of PAH exposure, which may underestimate the true impact on human health because high molecular weight PAHs are primarily excreted via bile rather than urine (21). For example, Yang et al. found that only 6.7% of pyrene (Pyr) entering the human body was excreted as hydroxylated metabolites, indicating that human PAH exposure may be underestimated (22). Evidence suggests that the PAH concentrations in the blood may be highly correlated with exposure, absorption, and retention (23). Population-based studies have measured plasma PAHs to reflect human exposure (23–25). Blood PAH levels have also been used to mimic environmental doses in studies of PAH-induced vascular endothelial toxicity (26). Therefore, there is an urgent need to explore associations between plasma PAH levels and platelet indicators, which may further enhance understanding of PAH exposure-related health effects and provide a basis for formulating public health policies.

Populations are widely exposed to PAH mixtures rather than individual PAHs. Given the intricate exposure patterns, high correlations, and complex interactions among environmental chemicals, it is necessary to evaluate the collective effects of various pollutants (27). In addition to the use of a generalized linear model, a quantile-based g calculation (QGC) model was used to evaluate the mixed effects of PAHs on platelet function. QGC has been widely used to assess the negative effects of multiple pollutants on human health (28, 29). Utilizing data from a subpopulation of the Henan Rural Cohort Study, and employing the analysis strategies outlined earlier, our study aimed to elucidate the associations between platelet indicators and exposure to individual PAHs and mixtures of PAHs. Furthermore, we sought to identify major influencing factors and the primary monomeric PAHs driving these associations.

## Materials and methods

### Study subjects

A total of 2,775 participants were selected from the Henan Rural Cohort Study in Henan Province, China, conducted between July 2015 and September 2017 (30). The data of participants were collected using questionnaires, basic physical examinations, and blood samples (31). All procedures were conducted in accordance with the principles of the Declaration of Helsinki. Ethical approval was obtained from the Life Science Ethics Committee of Zhengzhou University [Code: (2015) MEC (S128)], and all participants provided written informed consent before participating in this study. Sociodemographic data were collected using questionnaires. Current smokers were defined as study subjects who smoked at least one cigarette per day for more than 6 months. Smoking status was classified into three groups: current smokers, former smokers, and never smokers. Current drinkers were defined as study subjects who consumed alcohol 12 times or more in the past year. Drinking status was also classified into three groups: current drinkers, former drinkers, and never drinkers. The International Physical Activity Questionnaire was used to assess the physical activity of study subjects, and based on its recommended standards, physical activity was divided into three groups: low, moderate, and high. To collect daily cooking information, participants were asked the following questions: (1) “Do you cook at home regularly?” (2) “What type of cooking fuel do you primarily use?” Cooking fuel type was categorized as clean fuel (electricity, natural gas, or liquefied petroleum gas) or solid fuel (coal or wood). Kitchen ventilation conditions were categorized as non-ventilation (natural ventilation only) or ventilation (equipped with an exhaust hood or fan) (32). Subjects with missing data for Ant (*n* = 110), BaP (*n* = 18), PDW (*n* = 503), total lipid concentration (TLC) (*n* = 19), and cooking (*n* = 1) were excluded, and ultimately 2,124 subjects were included in the analysis, consisting of 797 men and 1,327 women.

### Measurement of the plasma PAHs

The plasma concentrations of 16 PAHs were measured, including six two- and three-ring PAHs [Naphthalene, Phenanthrene (Phe), Acenaphthene, Acenaphthylene, Fluorene, and Anthracene (Ant)] and 10 four- or higher-ring PAHs [Benz(a)anthracene, Benzo(a)pyrene (BaP), Benzo(b)fluoranthene (BbF), Benzo(g,h,i)perylene (BghiP), Benzo(k)fluoranthene (BkF), Chrysene (Chr), Dibenz(a,h)anthracene (DahA), Indeno(1,2,3-cd)pyrene (In123cdP), Fluoranthene (Flu), and Pyr]. Detection was performed using gas chromatography coupled to a triple quadrupole tandem mass spectrometry (GC-MS/MS). Briefly, 300 μL of plasma sample and 50 μL of 10 ppb internal standard (Phenanthrene-d10 or Perylene-d12) were mixed into glass tubes with 300 μL of Milli-Q water. The detailed method for extracting and separating 16 PAHs in plasma is described elsewhere (33). During this experiment, each batch (12 samples) included a blank control (all conditions except for water) and a quality control (adding PAH standard solution to fetal bovine serum) to monitor potential contamination. The limit of detection (LOD) of PAHs in plasma was defined as a signal-to-noise ratio greater than three. The detection limits of PAHs in plasma ranged from 0.003 to 0.078 ng/mL. Spike recoveries ranged from 80% to 143%, with relative standard deviations ranging from 5.32% to 12.00%. For PAH concentrations below the LOD, values were set to half the LOD. A total of 11 PAHs were detected in the plasma samples. Due to the high spiked recovery of In123cdP, only 10 PAHs with recoveries between 80% and 120% were included in this study as target analytes. Quantification of each compound was performed using linear regression curves (*R*^2^ ≥ 0.999) from the calibration of the corresponding internal standards. Detailed instrument parameters and detection results of PAHs are reported in a previously published article (33). The TLC was calculated using the following formula: total cholesterol (mmol/L) × 2.27 × 38.67 + triglyceride (mmol/L) × 88.54 + 62.3 (34). The plasma PAHs were adjusted by TLC to account for variation in plasma lipid levels.

### Platelet indicator measurement

All participants were enrolled and recruited through local community health examination centers. Venous blood samples were collected from participants in dipotassium-ethylenediaminetetraacetic acid tubes by trained nurses for routine blood analysis performed on the same day. Platelet parameters—including PLT, MPV, PDW, P-LCR, and PCT—were determined using an automated hematology analyzer (Sysmex XT-500i, Sysmex Corporation, Kobe, Japan). Additional platelet-based indicators included the MPV/PLT ratio, MPV/PCT, PDW/PLT, and PDW/PCT. A total of 10% samples were measured twice, with coefficients of variation <10%.

### Statistical analysis

In this study, mean and standard deviations were used to describe normally distributed data; median and interquartile ranges were used to describe continuous variables with non-normal distribution; and percentage and number were used to describe categorical variables. Student's *t*-test was used to compare the means of normally distributed variables between genders. The Mann–Whitney *U*-test was used to compare non-normally distributed continuous variables between genders. The chi-square test was used to compare the proportion of categorical variables between genders. Spearman's correlation analysis was used to assess correlations between different PAHs and evaluate collinearity. In this study, the independent association between PAHs and platelet-related indicators was explored using general linear models. We further applied the false discovery rate method to adjust *P*-values for multiple comparisons. The adjustment of covariates was based on univariate analysis (*P* < 0.10) and previous studies (16–18). Quantile g-calculation was used to assess the association between 10 detected PAH mixtures and platelet-related indicators, a method widely used to assess the health effects of environmental pollutant mixtures (35, 36). Furthermore, we used stratified analysis to assess the impact of different factors on the association between PAHs and platelet indicators. Data analyses were performed using the statistical package IBM-SPSS version 26.0 (IBM-SPSS Inc., Armonk, NY, USA) and R software version 4.2.1. The statistical significance level was set at a two-tailed *P*-value < 0.05.

## Results

### Basic characteristics of the study subjects

After excluding participants with missing values, the number of people included in the analysis decreased from 2,775 to 2,124, with 797 men and 1,327 women. Educational level, smoking status, drinking status, cooking status, high-fat diet, exercise, body mass index (BMI), total leukocyte count, hypertension, coronary heart disease, PLT, MPVP, MPV/PCT, PDW/PLT, and PDW/PCT were compared between men and women (*P* < 0.001). With the exception of PLT, which was higher in women, MPV, MPV/PCT, PDW/PLT, and PDW/PCT were all higher in men. The detailed results can be found in Table 1.

**Table 1:** Basic information on the characteristics of the study participants.

Variables	Total (*N* = 2,124)	Men (*N* = 797)	Women (*N* = 1,327)	*P*-value
Age (year, mean ± SD)	59.62 ± 8.81	59.72 ± 8.99	59.56 ± 8.71	0.691^a^
Education level (*n*, %)				<0.001^b^
Elementary school or below	1,178 (55.46)	311 (39.02)	867 (65.34)	
Middle school	745 (35.08)	358 (44.92)	387 (29.16)	
High school or above	201 (9.46)	128 (16.06)	73 (5.50)	
Married/living together (n, %)	245 (11.53)	92 (11.54)	153 (11.53)	1.000^b^
Average monthly income (*n*, %)				0.410^b^
<500 RMB	858 (40.40)	322 (40.40)	536 (40.39)	
500–999 RMB	649 (30.56)	232 (29.11)	417 (31.42)	
≥1,000 RMB	617 (29.05)	243 (30.49)	374 (28.18)	
Smoking status (yes, *n*, %)	525 (24.72)	521 (65.37)	4 (0.30)	<0.001^b^
Drinking status (yes, *n*, %)	378 (17.80)	351 (44.04)	27 (2.03)	<0.001^b^
Cooking status (yes, *n*, %)	1,571 (73.96)	313 (39.27)	1,258 (94.80)	<0.001^b^
High-fat diet (≥75 g/day, *n*, %)	402 (18.93)	185 (23.21)	217 (16.35)	<0.001^b^
Vegetable intake (≥500 g/day, *n*, %)	1,333 (62.76)	514 (64.49)	819 (61.72)	0.217^b^
Exercise (*n*, %)				<0.001^b^
Low	558 (26.27)	272 (34.13)	286 (21.55)	
Middle	1,033 (48.63)	275 (34.50)	758 (57.12)	
High	533 (25.09)	250 (31.37)	283 (21.33)	
BMI (kg/m^2^, mean ± SD)	24.64 ± 3.53	24.31 ± 3.31	24.83 ± 3.64	0.001^a^
TLC (mg/dL, median, IQR)	636.39 (555.49, 731.71)	606.67 (532.45, 699.31)	647.93 (572.41, 745.64)	<0.001^c^
History of diseases (*n*, %)				
T2DM	613 (28.86)	222 (27.85)	391 (29.46)	0.457^b^
HTN	626 (29.50)	182 (22.86)	444 (33.48)	<0.001^b^
CHD	154 (7.25)	36 (4.52)	118 (8.89)	<0.001^b^
Platelet-related indicators (median, IQR)				
PDW (fl)	15.40 (13.40, 17.90)	15.20 (13.20, 17.90)	15.50 (13.50, 17.90)	0.085^c^
PLT (10^9^/L)	196 (157, 242)	183 (145, 219)	206 (166, 254)	<0.001^c^
MPV (fl)	11.80 (10.80, 12.80)	11.70 (10.80, 12.80)	11.90 (10.90, 12.80)	0.085^c^
P-LCR	39.00 (32.00, 46.80)	38.20 (31.60, 46.40)	39.80 (32.40, 47.00)	0.034^c^
MPVP	0.06 (0.05, 0.08)	0.06 (0.05, 0.08)	0.06 (0.04, 0.08)	<0.001^c^
MPV/PCT	51.20 (41.39, 63.78)	54.71 (46.00, 68.89)	48.70 (39.33, 60.42)	<0.001^c^
PDW/PLT	0.08 (0.06, 0.11)	0.08 (0.06, 0.12)	0.08 (0.05, 0.11)	<0.001^c^
PDW/PCT	66.52 (52.00, 88.32)	71.11 (56.45, 95.56)	63.75 (49.58, 83.33)	<0.001^c^

a Student's *t-*test was used to compare normally distributed continuous variables between genders.

b Chi-square test was used to test the distribution of categorical variables between genders.

c Kruskal–Wallis *H*-test was used to compare non-normal distributed variables between genders.

SD, standard deviation; RMB, renminbi; BMI, body mass index; TLC, total lipid concentration; PDW, platelet volume distribution width; PLT, platelet count; MPV, mean platelet volume; P-LCR, platelet large cell ratio; MPVP, ratio of mean platelet volume to platelet count; PCT, thrombocytocrit; T2DM: type 2 diabetes mellitus; HTN, hypertension; CHD, chronic coronary heart disease.

The results of correlation analysis of platelet-related indicators are shown in Supplementary Figure S1. Among the positive correlations, the weakest correlations were observed between MPV/PCT and PDW (*r* = 0.62), between MPV/PCT and P-LCR (*r* = 0.62), and between MPV/PCT and MPV (*r* = 0.63). The other positive associations were strong. Among the negative correlations, the weakest correlations were observed between PDW and PLT (*r* = −0.62), between PDW and P-LCR (*r* = −0.62), and between PLT and MPV (*r* = −0.63).

### Distribution of plasma PAHs

**Figure 1:**
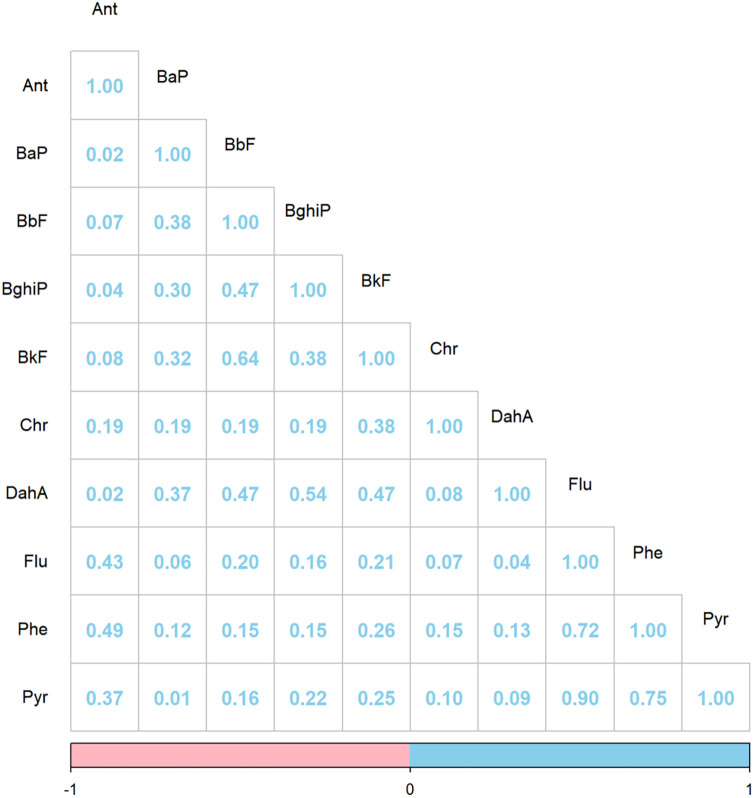
Spearman's correlation coefficient of PAHs. PAHs, polycyclic aromatic hydrocarbons; Ant, Anthracene; BaP, Benzo(a)pyrene; BbF, Benzo(b)fluoranthene; BghiP, Benzo(g,h,i)perylene; BkF, Benzo(k)fluoranthene; Chr, Chrysene; DahA, Dibenz(a,h)anthracene; Flu, Fluoranthene; Phe, Phenanthrene; Pyr, Pyrene.

Table 2 presents the distribution of PAHs in the participants' blood, with detection rates for 10 PAHs above 65%. The top three PAHs with the highest blood concentration were Phe, Ant, and Pyr. The original median and corrected median were 7.00 μg/L (2.36–17.99 μg/L) and 10.67 μg/g (3.46–28.85 μg/g) for Phe, 0.98 μg/L (0.51–2.29 μg/L) and 1.48 μg/g (0.76–3.67 μg/g) for Ant, and 0.53 μg/L (0.19–1.02 μg/L) and 0.81 μg/g (0.26–1.65 μg/g) for Pyr. The original median and the corrected median of BaP, the PAH with the lowest concentration in blood, were 0.11 μg/L (0.07–0.16 μg/L) and 0.17 μg/g (0.11–0.27 μg/g). The results of the correlation analysis are shown in Figure 1. The closer the absolute value of Spearman's correlation coefficient to 1, the stronger the correlation between the two factors. A correlation coefficient of 0.7 indicated strong correlation between the two factors, 0.4–0.7 indicated moderate correlation, and <0.4 indicated low correlation. The strongest correlation was between Flu and Pyr at 0.90, followed by Phe and Pyr at 0.75 and Flu and Phe at 0.72.

**Table 2:** Distribution of individual PAH exposure.

PAHs	LOD	Detection rate (%)	Original (μg/L)		Corrected (μg/mg lipid)	
			Median	IQR	Median	IQR
Ant	0.078	95.15	0.98	0.51–2.29	1.48	0.76–3.67
BaP	0.013	98.26	0.11	0.07–0.16	0.17	0.11–0.27
BbF	0.017	99.53	0.23	0.14–0.33	0.34	0.22–0.52
BghiP	0.004	99.95	0.16	0.11–0.20	0.24	0.15–0.34
BkF	0.018	96.47	0.14	0.10–0.19	0.21	0.15–0.31
Chr	0.006	99.58	0.15	0.08–0.25	0.23	0.12–0.40
DahA	0.003	99.95	0.18	0.10–0.29	0.27	0.16–0.45
Flu	0.028	66.29	0.35	0.01–1.07	0.51	0.02–1.71
Phe	0.040	84.32	7.00	2.36–17.99	10.67	3.46–28.85
Pyr	0.005	77.59	0.53	0.19–1.02	0.81	0.26–1.65

PAHs, polycyclic aromatic hydrocarbons; Ant, Anthracene; BaP, Benzo(a)pyrene; BbF, Benzo(b)fluoranthene; BghiP, Benzo(g,h,i)perylene; BkF, Benzo(k)fluoranthene; Chr, Chrysene; DahA, Dibenz(a,h)anthracene; Flu, Fluoranthene; Phe, Phenanthrene; Pyr, Pyrene; LOD, limit of detection; IQR, interquartile range.

### Association between individual PAH concentrations and platelet indicators

**Figure 2:**
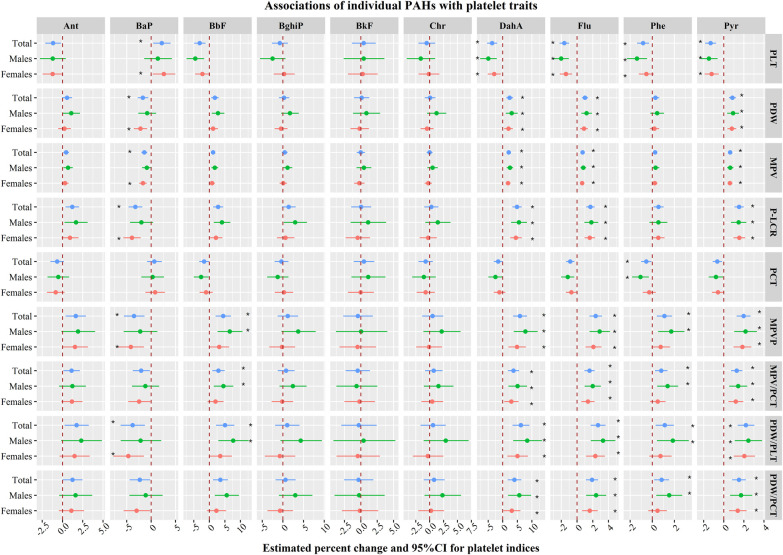
Associations between individual PAHs and platelet-related indicators. PDW, platelet volume distribution width; PLT, platelet count; MPV, mean platelet volume; P-LCR, platelet large cell ratio; MPVP, ratio of mean platelet volume to platelet count; PCT, thrombocytocrit; Ant, Anthracene; BaP, Benzo(a)pyrene; BbF, Benzo(b)fluoranthene; BghiP, Benzo(g,h,i)perylene; BkF, Benzo(k)fluoranthene; Chr, Chrysene; DahA, Dibenz(a,h)anthracene; Flu, Fluoranthene; Phe, Phenanthrene; Pyr, Pyrene. The model was adjusted for BMI, educational level, marital status, average monthly income, smoking status, drinking status, cooking status, high-fat diet, fruit and vegetable intake, and history of T2DM, HTN, and CHD.

Smoking women (*n* = 4) and non-cooking women (*n* = 69) were excluded from these models. As shown in Figure 2, among all participants, the estimated percent change and 95% confidence interval (CI) in PLT for each unit increment in naturally transformed Ant, BbF, DahA, Flu, Phe, Pyr, and BaP were −1.2% (−2.1, −0.2%), −3.1% (−4.9, −1.3%), −3.6% (−5.4, −1.9%), −1.6% (−2.2, −1%), −0.8% (−1.4, −0.3%), −1.3% (−1.9, −0.8%), and 2.2% (0.4, 4%). Positive associations were observed of BbF, DahA, Flu, Phe, and Pyr with platelet size indices, but BaP showed negative associations of with platelets. For instance, the estimated percent change and 95% CI of MPV in response to each unit increment in naturally transformed BbF, DahA, Flu, Phe, Pyr, and BaP were 2.8% (1.2, 4.5%), 4.9% (3.3, 6.5%), 1.7% (1.2, 2.2%), 0.6% (0.1, 1%), 1.5% (1.1, 2%), and −3.2% (−4.6, −1.8%). Similar results were observed across genders.

### Association between PAH mixtures and platelet indicators

Smoking women (*n* = 4) and non-cooking women (*n* = 69) were excluded from these models. Table 3 and Supplementary Figure S1 present the association between each quantile increase of PAH mixtures and platelet indicators, with the weights of each PAH estimated by QGC models. Results from the QGC models showed that the estimated percent change and 95%CI of PLT, PDW, MPV, P-LCR, PCT, MPVP, MPV/PCT, PDW/PLT, and PDW/PCT for each quantile increment in PAH mixtures were −6.1% (−8.5, −3.6%), 2.7% (1.1, 4.4%), 2.2% (1.3, 3.1%), 5.5% (3.3, 7.7%), −4.0% (−6.1, −1.8%), 8.8% (5.4, 12.3%), 6.4% (3.6, 9.2%), 9.4% (5.3, 13.6%), and 7.0% (3.6, 10.4%) after adjusting for covariates. These results of the association between PAH mixtures and platelet indices did not show substantial changes across genders. As shown in Supplementary Figure S1, DahA and Flu were the major contributors to the observed associations of PAH mixtures with platelet indices, accounting for nearly 50% of the mixture weights.

**Table 3:** The association between PAH mixtures and platelet-related indicators among all participants.

Variables	Total population	Males	Females
	Estimated percent change (95% CI)	Estimated percent change (95% CI)	Estimated percent change (95% CI)
PLT	−6.1 (−8.5, −3.6)	−8.0 (−11.8, −4)	−5.3(−8.3, −2.2)
PDW	2.7 (1.1, 4.4)	4.4 (1.6, 7.4)	1.8 (0.7, 2.9)
MPV	2.2 (1.3, 3.1)	3.0 (1.5, 4.6)	2.2 (1.3, 3.1)
P-LCR	5.5 (3.3, 7.7)	7.2 (3.4, 11.1)	4.9 (2.2, 7.7)
PCT	−4.0 (−6.1, −1.8)	−4.9 (−8.2, −1.5)	−3.6 (−6.4, −0.8)
MPVP	8.8 (5.4, 12.3)	12.0 (6.2, 18)	7.5 (3.3, 11.9)
MPV/PCT	6.4 (3.6, 9.2)	8.4 (3.8, 13.1)	5.6 (2.1, 9.3)
PDW/PLT	9.4 (5.3, 13.6)	13.5 (6.5, 21)	7.7 (2.7, 13)
PDW/PCT	7.0 (3.6, 10.4)	9.9 (4.3, 15.8)	5.9 (1.7, 10.2)

PDW, platelet volume distribution width; PLT, platelet count; MPV, mean platelet volume; P-LCR, platelet large cell ratio.

The model was adjusted for age, BMI, educational level, marital status, average monthly income, drinking status, cooking status, high-fat diet, fruit and vegetable intake, and history of T2DM, HTN, and CHD.

### Stratified analysis of associations of PAH levels with platelet indicators

Detailed results are presented in Table 4 and Supplementary Figure S2. As shown in Supplementary Figure S2, positive associations of DahA and Flu with platelet size indices of PDW, MPV, P-LCR, MPVP, MPV/PCT, PDW/PLT, and PDW/PCT were found, while negative associations with PLT were found among smoking men, non-smoking men, and non-smoking women. As shown in Table 4, the estimated percent changes in platelet indices of PLT, PDW, MPV, P-LCR, PCT, MPVP, MPV/PCT, PDW/PLT, and PDW/PCT in response to each quantile increment in PAH mixtures were −8.3% (−12.7, −3.6%), 3.2% (−0.3, 6.8%), 2.4% (0.5, 4.3%), 5.7% (1.1, 10.5%), −5.8% (−9.6, −1.8%), 11.7% (4.9, 18.9%), 8.7% (3.4, 14.3%), 12.5% (4.3, 21.5%), and 9.5% (2.9, 16.5%) among smoking men, after adjusting for covariates. Similar results were observed among non-smoking men and women. As shown in Supplementary Figure S3, positive associations were observed between DahA and Flu and the platelet size indices of PDW, MPV, MPV/PCT, PDW/PLT, and PDW/PCT, with negative associations for PLT among smoking men, non-smoking men and non-smoking women. As shown in Table 5, the estimated percent changes in platelet indices of PLT, PDW, MPV, P-LCR, PCT, MPVP, MPV/PCT, PDW/PLT, and PDW/PCT in response to each quantile increment in PAH mixtures were −13.4% (−22.5, −3.3%), 8.4% (0.7, 16.6%), 3.5% (0.7, 6.3%), 10.1% (−0.1, 21.3%), −8.8% (−16.8, 0.1%), 20.6% (5.0, 38.6%), 14.5% (2.3, 28.1%), 5.2% (6.0, 47.9%), and 18.8% (3.6, 36.3%) among non-smoking and non-cooking men, after adjusting for covariates. Similar results were observed among non-smoking women who reported self-cooking. The estimated percent change in MPV in response to each quantile increment in PAH mixtures was 2.4% (0.5, 4.3%) among non-smoking men with self-cooking. Stratified analyses were further performed based on cooking-related behaviors. As shown in Supplementary Figure S4, estimated percent changes in platelet indices associated with each unit increment in plasma PAH values were lower among participants who cooked using mechanical ventilation than among those who cooked using natural ventilation. Such estimated percent changes were higher among participants who cooked using solid fuel than among those who cooked using clean fuel.

**Table 4:** The association between PAH mixtures and platelet-related indicators among all participants.

Variables	Smoking males	Non-smoking males	Non-smoking females
	Estimated percent change (95% CI)	Estimated percent change (95% CI)	Estimated percent change (95% CI)
PLT	−8.3 (−12.7, −3.6)	−7.5(−14.8, 0.4)	−5.2 (−8.3, −2.1)
PDW	3.2 (−0.3, 6.8)	5.7 (0.5, 11.2)	2.1 (0, 4.2)
MPV	2.4 (0.5, 4.3)	3.5 (0.7, 6.3)	1.8 (0.7, 3)
P-LCR	5.7 (1.1, 10.5)	8.7 (1.8, 16.1)	5.0 (2.2, 7.8)
PCT	−5.8 (−9.6, −1.8)	−4.1 (−10.5, 2.8)	−3.5 (−6.3, −0.7)
MPVP	11.7 (4.9, 18.9)	11.9 (1.1, 23.9)	7.4 (3.2, 11.9)
MPV/PCT	8.7 (3.4, 14.3)	7.9 (−0.8, 17.2)	5.6 (2.1, 9.2)
PDW/PLT	12.5 (4.3, 21.5)	14.3 (1.2, 29.2)	7.7 (2.7, 12.9)
PDW/PCT	9.5 (2.9, 16.5)	10.2 (−0.5, 22)	5.8 (1.6, 10.1)

PDW, platelet volume distribution width; PLT, platelet count; MPV, mean platelet volume; P-LCR, platelet large cell ratio.

The model was adjusted for age, BMI, educational level, marital status, average monthly income, drinking status, cooking status, high-fat diet intake, vegetables intake, and history of T2DM, HTN, and CHD.

**Table 5:** The association between PAH mixtures and platelet-related indicators among all participants.

Variables	Non-smoking males with self-cooking	Non-smoking and self-cooking males	Non-smoking females with self-cooking
	Estimated percent change and 95% CIs	Estimated percent change and 95% CIs	Estimated percent change and 95% CIs
PLT	−0.3 (−11.5, 12.3)	−13.4 (−22.5, −3.3)	−4.8 (−7.9, −1.5)
PDW	3.4 (−3.7, 11.0)	8.4 (0.7, 16.6)	1.8 (−0.3, 3.9)
MPV	2.4 (0.5, 4.3)	3.5 (0.7, 6.3)	1.8 (0.7, 3)
P-LCR	6.4 (−2.8, 16.6)	10.1 (−0.1, 21.3)	4.6 (1.8, 7.5)
PCT	1.9 (−7.8, 12.6)	−8.8 (−16.8, 0.1)	−3.3 (−6.1, −0.4)
MPVP	2.8 (−11.2, 18.9)	20.6 (5, 38.6)	6.8 (2.5, 11.3)
MPV/PCT	0.5 (−10.8, 13.3)	14.5 (2.3, 28.1)	5.1 (1.6, 8.8)
PDW/PLT	3.7 (−12.9, 23.4)	25.2 (6.0, 47.9)	6.9 (1.8, 12.3)
PDW/PCT	1.4 (−12.3, 17.3)	18.8 (3.6, 36.3)	5.2 (1.0, 9.7)

PDW, platelet volume distribution width; PLT, platelet count; MPV, mean platelet volume; P-LCR, platelet large cell ratio. The model was adjusted for age, BMI, educational level, marital status, average monthly income, drinking status, high-fat diet intake, vegetables intake, and history of T2DM, HTN, and CHD.

## Discussions

This study found that PAH exposure was associated with platelet function indicators, suggesting that exposure to PAHs was related to increased platelet size indicators. Those effects were prominent among smokers and women engaging in self-cooking in rural regions. Automobile usage in China is becoming increasingly widespread, and the effects on PAH emission cannot be ignored (37, 38). Evidence suggests that PAHs in PM are primarily derived from coal combustion and automobile exhaust (38). A cross-sectional study of urban Chinese adults showed that cigarette smoking, self-cooking, and prolonged time spent in traffic were associated with increased urinary PAH metabolites (39). Furthermore, Zhang et al. reported that cooking is a critical factor affecting personal PAH exposure to PM among rural residents in Northern China (40). Peng et al. suggested that the proportion of smoking among the rural population was higher than that in the urban population (41). Taken together, we may infer that women and smokers are more sensitive to the negative effects of PAH exposure on platelet activity, partly due to smoking and cooking emissions.

Platelets have been considered blood biomarkers of inflammation and immune responses (12). MPV and PDW have been associated with incident CVDs, as they reflect the activity of platelets in thrombosis and inflammation (42). In the present study, certain plasma PAHs were associated with increased platelet size indicators. Previous research has investigated the effect of PAHs on platelet activation, reporting synergy between Ant and Pyr, whereas Chr, BaP, and BghiP showed inhibitory effects (43). Yuan et al. conducted a cross-sectional study and found nonlinear associations between urinary PAH metabolites and increased MPV, PDW, and MPVP among Chinese urban adults (18). Dai et al. demonstrated that exposure to PAHs was positively associated with PLT and P-LCR, and negatively associated with MPVP (17). Limited evidence also suggests that PAH exposure is associated with elevated platelet counts, with sex and age modulating the association of hydroxyphenylalanine and 1-OHP with inflammatory biomarkers, due to which girls and younger children were more affected (20). In addition, associations between PM pollution and platelet indicators have not been consistent (44–46). For instance, results from the Heinz Nixdorf Recall Study showed that a positive association between increased PM_2.5_ concentration and increased PLT was observed among 4,814 German adults (44). Results from the Henan Rural Cohort Study showed positive associations between PM of different sizes and platelet size indicators (46). Moreover, genetic polymorphisms such as CYP1A1 and CYP2E1 may affect PAH metabolites in relation to smoking (47). The inconsistent results between this study and previous studies may be partly attributed to the differences in study regions and designs, PAH concentrations and measurement methods, as well as heterogeneity in study populations.

Humans are often exposed to multiple PAHs in the environment; therefore, it is both important and urgent to evaluate the joint association between the multiple PAHs and platelet function indicators. In this study, the results of QGC showed positive joint associations of PAHs with platelet size indicators. Few studies have assessed multi-PAH exposure in relation to platelet function. For instance, Hu et al. applied structural equation modeling to reveal that urinary PAH metabolites were positively associated with MPV among Chinese adults (48). Abulikemu et al. applied QGC models to reveal that platelet parameters mediated associations between PAH exposure and blood pressure alterations among 558 workers in a cooking plant (49). Rural residents often inhale PAHs through the respiratory tract, with long-term low-dose exposure arising from field burning, smoking, and cooking, leading to cumulative composite exposure. Factors such as high smoking rates, poor kitchen ventilation, and frequent burning greatly increase the total exposure load compared with single sources, essentially reflecting the release of the same chemicals across different scenarios (50–52).

This study has several limitations. First, this is a cross-sectional study that was meant to explore the relationship between PAH exposure and platelet traits, but the causal relationship between them could not be established. Therefore, the results of this study should be confirmed with a prospective study. Second, the investigators may have introduced recall bias during the investigation, due to the fact that information on cooking and smoking behaviors was collected using questionnaires. Third, selection bias may have occurred because the study population primarily consisted of elderly participants. Thus, the results of this study are limited and cannot be generalized to other populations, and interpretation should be made with caution. Fourth, although we controlled for several important factors, other environmental factors that may influence the results were not considered, such as traffic-related PAHs (39, 53).

## Conclusions

This study's findings show that long-term exposure to low concentrations of PAHs is associated with increased platelet size, with effects differing between male and female populations. Furthermore, positive associations between mixtures of 10 PAHs and platelet indicators—except for PLT—were observed among women who engaged in self-cooking. These results suggest that mitigating the negative effects on platelet function may be achieved by prioritizing PAH emission control and improving kitchen ventilation, particularly for women who cook regularly.

## Data Availability

The raw data supporting the conclusions of this article will be made available by the authors, without undue reservation.
