# Supplementary material for: Differential associations of cooking behaviors with polycyclic aromatic hydrocarbon exposure-related platelet traits as cardiovascular risk biomarkers
Source: Front Cardiovasc Med. 2026 May 15;13:1703901. doi: 10.3389/fcvm.2026.1703901 (PMC13218849; doi:10.3389/fcvm.2026.1703901)
Supplement: Supplementary file 1 [file Table1.docx]

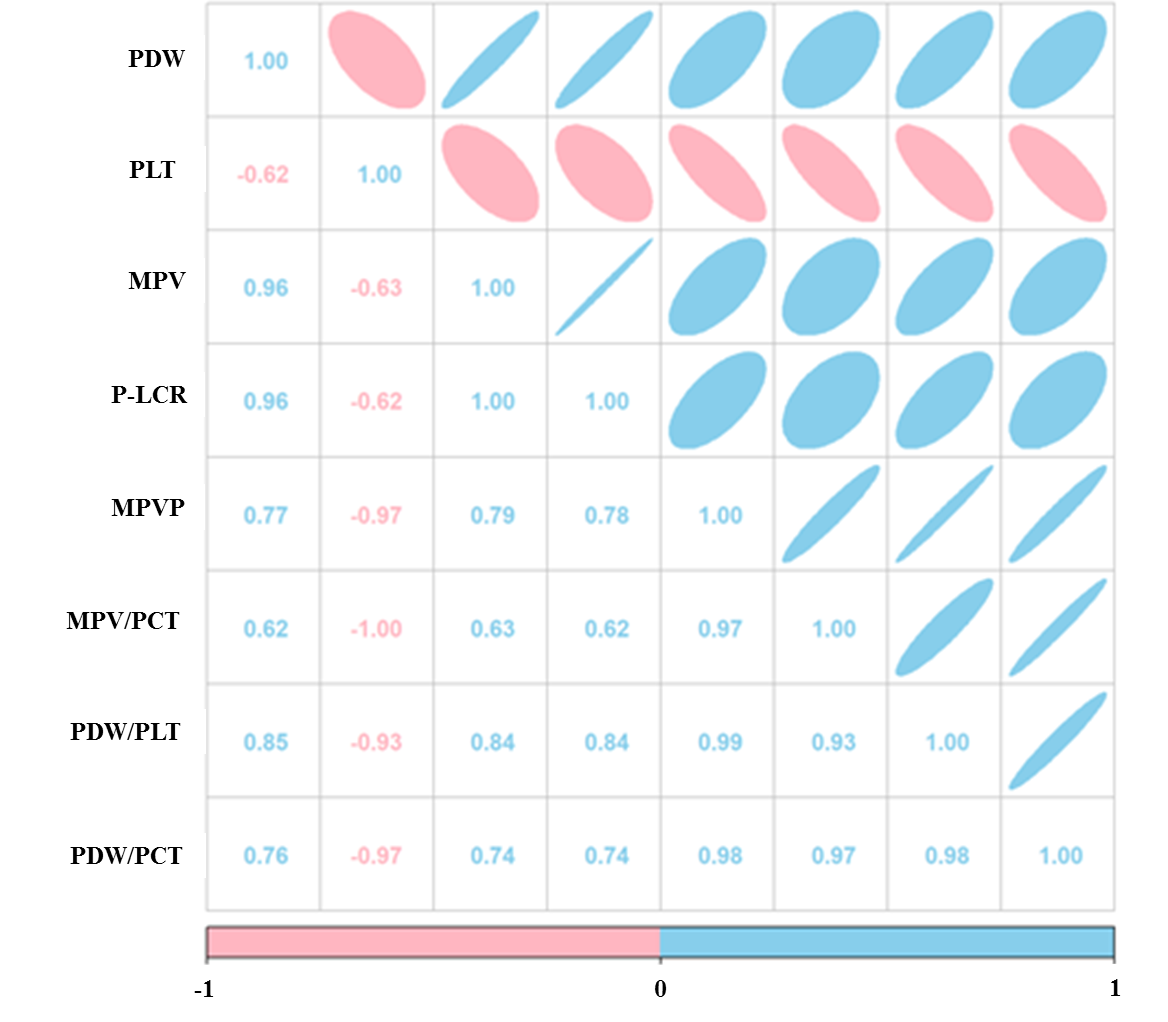


Supplementary fig 1 Spearman's correlation coefficient of platelets-related indicators.


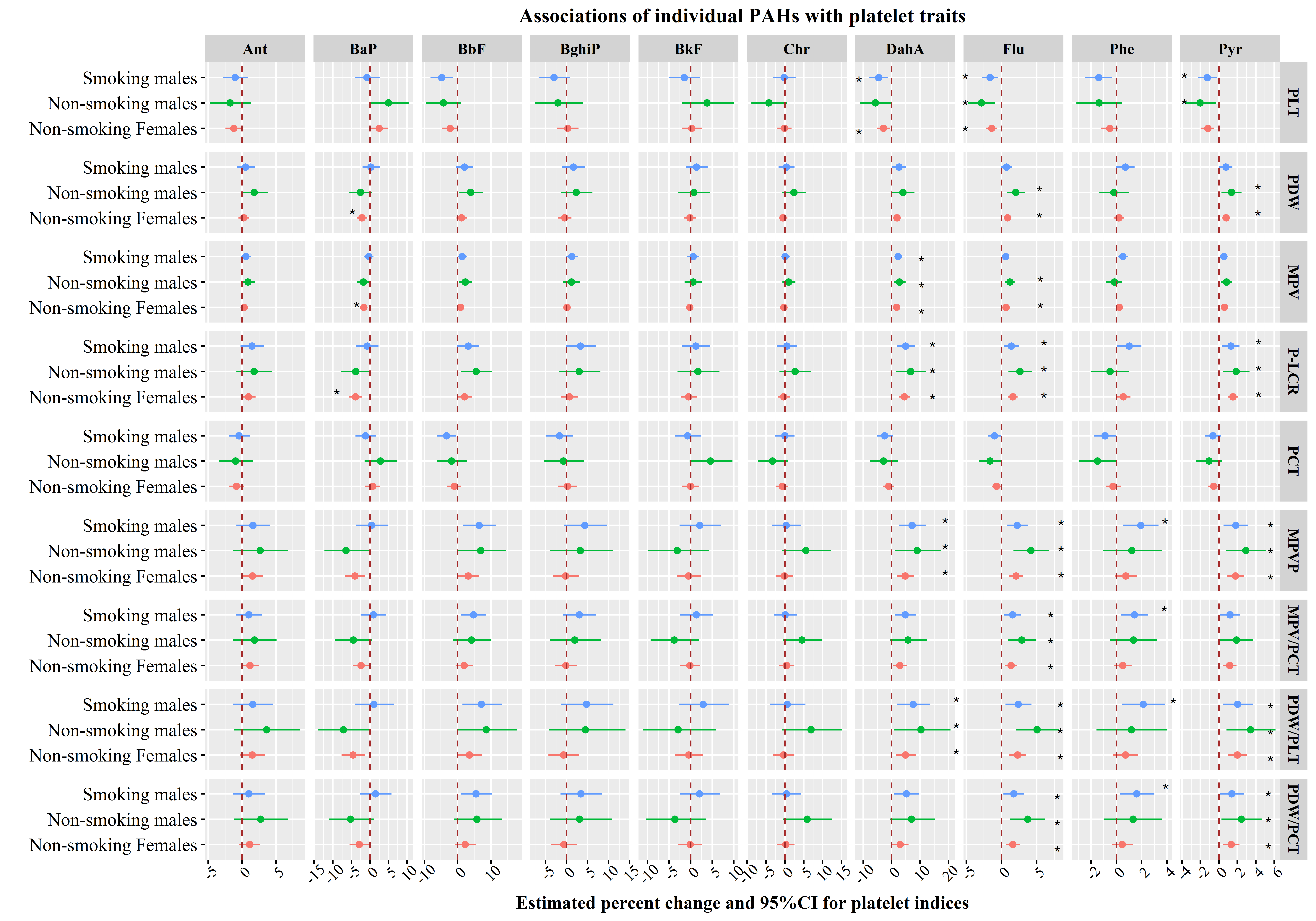
Supplementary fig 2 Associations of individual PAH with platelet-related indicators.

Abbreviations: PDW: platelet volume distribution width; PLT: platelet count; MPV: mean platelet volume; P-LCR: platelet large cell ratio; MPVP: ratio of mean platelet volume to platelet count; PCT: thrombocytocrit; Ant: Anthracene; BaP: Benzo(a)pyrene; BbF: Benzo(b)fluoranthene; BghiP: Benzo(g,h,i)perylene; BkF: Benzo(k)fluoranthene; Chr: Chrysene; DahA: Dibenz(a,h)anthracene; Flu: Fluoranthene; Phe: Phenanthrene; Pyr: Pyrene.

The model was adjusted for BMI, educational level, marital status, average monthly income, drinking status, cooking status, high fat diet intake, vegetables intake and history of T2DM, HTN and CHD.


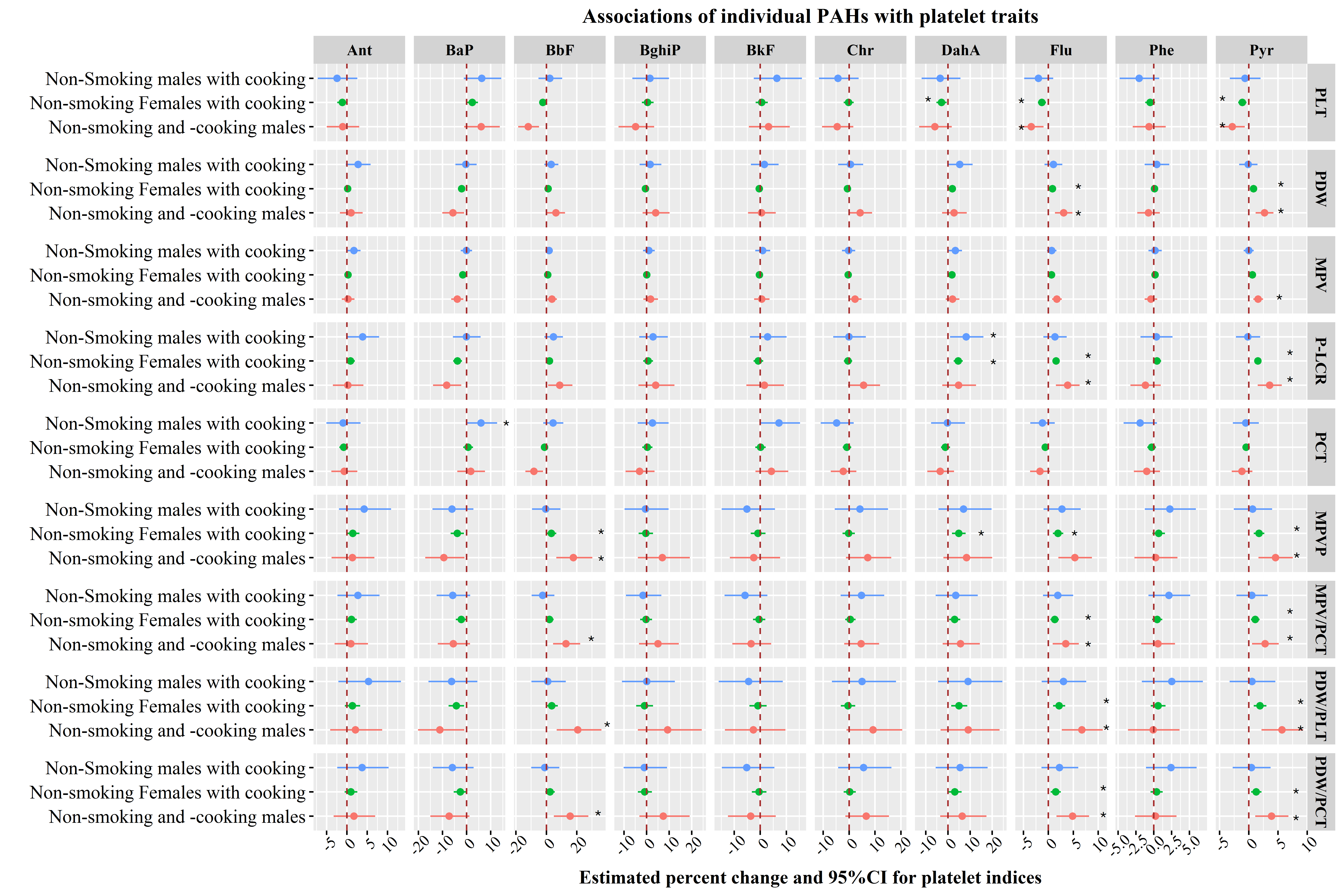


Supplementary fig 3 Associations of individual PAH with platelet-related indicators.

Abbreviations: PDW: platelet volume distribution width; PLT: platelet count; MPV: mean platelet volume; P-LCR: platelet large cell ratio; MPVP: ratio of mean platelet volume to platelet count; PCT: thrombocytocrit; Ant: Anthracene; BaP: Benzo(a)pyrene; BbF: Benzo(b)fluoranthene; BghiP: Benzo(g,h,i)perylene; BkF: Benzo(k)fluoranthene; Chr: Chrysene; DahA: Dibenz(a,h)anthracene; Flu: Fluoranthene; Phe: Phenanthrene; Pyr: Pyrene.

The model was adjusted for BMI, educational level, marital status, average monthly income, drinking status, high fat diet intake, vegetables intake and history of T2DM, HTN and CHD.


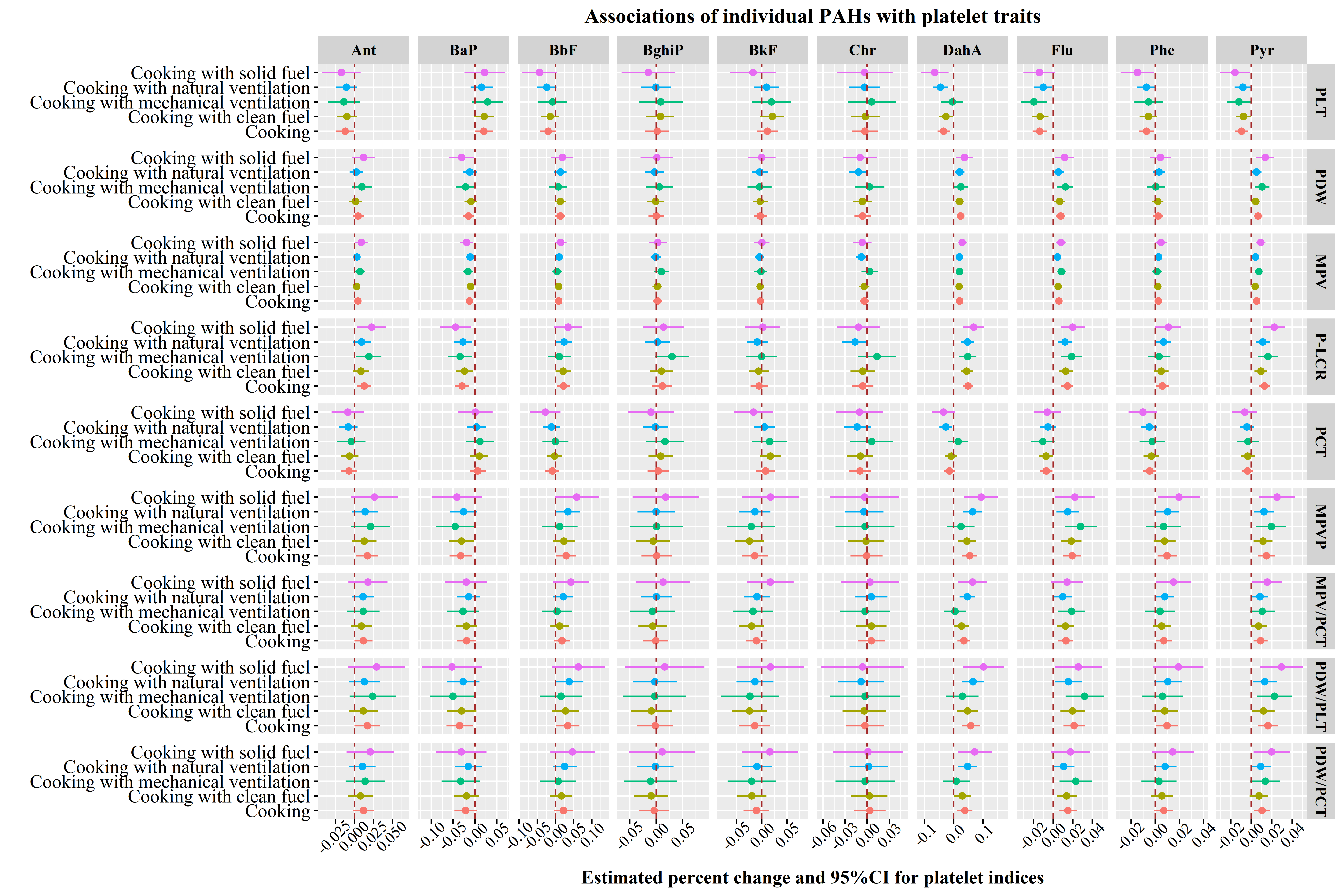


Supplementary fig 4 Associations of individual PAH with platelet-related indicators.

Abbreviations: PDW: platelet volume distribution width; PLT: platelet count; MPV: mean platelet volume; P-LCR: platelet large cell ratio; MPVP: ratio of mean platelet volume to platelet count; PCT: thrombocytocrit; Ant: Anthracene; BaP: Benzo(a)pyrene; BbF: Benzo(b)fluoranthene; BghiP: Benzo(g,h,i)perylene; BkF: Benzo(k)fluoranthene; Chr: Chrysene; DahA: Dibenz(a,h)anthracene; Flu: Fluoranthene; Phe: Phenanthrene; Pyr: Pyrene.

Cooking model was adjusted for age, BMI, gender, educational level, marital status, average monthly income, drinking status, smoking status, exercise, high fat diet intake, fuel type, ventilation, cooking duration, vegetables intake and history of T2DM, HTN and CHD.

Associations of PAHs exposure with platelet-related indicators stratified by cooking fuel types were adjusted for age, BMI, gender, educational level, marital status, average monthly income, drinking status, smoking status, exercise, high fat diet intake, kitchen ventilation, cooking duration, vegetables intake and history of T2DM, HTN and CHD.

Associations of PAHs exposure with platelet-related indicators stratified by kitchen ventilation conditions were adjusted for age, BMI, gender, educational level, marital status, average monthly income, drinking status, smoking status, exercise, high fat diet intake, fuel type, cooking duration, vegetables intake and history of T2DM, HTN and CHD.
